# Efficacy of the Synergy Between Live-Attenuated and Inactivated PRRSV Vaccines Against a NADC30-Like Strain of Porcine Reproductive and Respiratory Syndrome Virus in 4-Week Piglets

**DOI:** 10.3389/fvets.2022.812040

**Published:** 2022-02-02

**Authors:** Chaosi Li, Zhicheng Liu, Kai Chen, Jie Qian, Yulong Hu, Shuhe Fang, Zhi Sun, Chunhong Zhang, Lv Huang, Jianfeng Zhang, Nian Huang

**Affiliations:** ^1^Boehringer Ingelheim Animal Health (Shanghai) Co. Ltd., Shanghai, China; ^2^Maoming Branch Center of Guangdong Laboratory for LingNan Modern Agricultural Science and Technology, Key Laboratory of Livestock Disease Prevention of Guangdong Province, Scientific Observation and Experiment Station of Veterinary Drugs and Diagnostic Techniques of Guangdong Province, Ministry of Agriculture and Rural Affairs, Institute of Animal Health, Guangdong Academy of Agricultural Sciences, Guangzhou, China; ^3^Asian Veterinary Research and Development Center, Boehringer Ingelheim (China) Investment Co., Ltd., Shanghai, China

**Keywords:** porcine reproductive and respiratory syndrome virus, NADC30-like, vaccination, heterologous-protection, pathogenicity, inactivated vaccine

## Abstract

The NADC30-like strain of porcine reproductive and respiratory syndrome virus (PRRSV) is a novel strain responsible for substantial economic losses to swine production in China. This study evaluated the cross-protective efficacy of the synergy between live-attenuated and inactivated PRRSV vaccines compared with a single vaccination with PRRS modified-live virus (MLV) vaccine against challenge with NADC30-like strain, v2016/ZJ/09-03. A total of 45 PRRSV free pigs were randomly divided into five groups: (1) strict control (SC); (2) positive control (PC); (3) single MLV dose (M1); (4) primed intramuscularly with MLV and boosted with killed vaccine 3 weeks later (MK1); and (5) intramuscular prime MLV boosted subcutaneously with killed vaccine B 3 weeks later (MK2). Serological tests in MK groups revealed no differences in both anti-N and anti-GP protein antibodies compared with M1 group, and failed to provide further protection against clinical signs, virus shedding, and gross lesions. However, the viremic titer, gross lung lesions, and average daily weight gain were significantly improved in the MLV vaccinated groups, suggesting that MLV provides substantial cross-protection against the NADC30-like virus. Thus, as a booster, the killed vaccine confers minimal additional protection in NADC30-like infected piglets.

## Introduction

Porcine reproductive and respiratory syndrome (PRRS) was first reported in North America in 1987 (1) and was subsequently identified in both Europe and Asia in the 1990s (2–5). Porcine reproductive and respiratory syndrome virus (PRRSV) is an enveloped, single positive-stranded RNA virus that belongs to the family *Arteriviridae*, genus *Betaarterivirus*, and consists of two genotypes: PRRSV-1 (European) and PRRSV-2 (North American). In 1991, the European prototypic strain, Lelystad, was first isolated in the Netherlands. The following year, the North American prototypic strain, VR2332, was isolated in the United States (6, 7). In China, there have been three main prevalence stages, each of which has been associated with its own dominant representative strain and lasts for ~10 years. PRRS was first described in China in 1995, followed by the etiological agent, CH-1a (GenBank ID: AY032626) and BJ-4 (GenBank ID: AF331831), isolated by Guo et al. (8) in 1996 and Yang et al. (9) in 1997, respectively. Both strains belong to PRRSV-2. In the summer of 2006, a novel highly pathogenic PRRSV variant strain (HP-PRRSV) characterized by high mortality, hyperpyrexia, and a high abortion rate resulted in devastating destruction to the swine industry (10). Genetic analyses have revealed that HP-PRRS shows a unique deletion in 30 discontinuous amino acids (482 aa, 534–562 aa) in the Nsp2 gene (11). After 2006, HP-PRRSV strains become the predominant epidemic strains on pig farms (12). Since 2014, NADC30-like strains gradually increased in dominancy from HP-PRRS and became the most prevalent strains in mainland China (13, 14). Liu et al. (14) reported that from 2017 to 2019, 39 out of 62 PRRS positive fecal swabs were the NADC30-like strain.

PRRS is a devastating disease for pork producers as it causes reproductive failure in sow herds, extending unproductive days and respiratory disorders in growing to fatting herds, resulting in increased culling and mortality rates (15). Moreover, PRRS also causes other losses (e.g., feed intake, additional medication expenses, and labor costs) to the whole swine industry (16). An economic calculator found that the impact of PRRS on farm profits was −19.1% on average and −41% as a worst case (17). One study employed an epidemiological and economic disease model to determine the costs of PRRSV on pig farms, and found that the losses were not often obvious, ranging from $87,499 to $751,179 annually for individual farrow-to-finish farms (1,000 sows) depending on the degree to which the herd was affected by PRRS (18).

Since vaccines represent one of the main tools for improving animal health and for reducing or limiting pathogen transmission, PRRS vaccine research and development was rapidly initiated. In 1994, a PRRSV-2 modified-live virus (MLV) vaccine was first commercialized in North America, and 6 years later, a PRRSV-1 MLV vaccine was also licensed in Europe (19). Currently, a “perfect” PRRS vaccine has not been successfully developed (20). All commercial vaccines are primarily categorized by either MLV or killed virus (KV) vaccines, each has its own pros and cons (20–24). Although safety is the main advantage of the KV vaccine, it confers limited or rare efficacy against homologous and heterologous viruses, particularly, in naïve animals (23, 25). In contrast, MLV vaccines can confer complete homologous-protection and partial heterologous-protection (21, 22, 25). Therefore, MLV vaccination has been the principal intervention method used to reduce the economic loss during PRRSV infection and has gradually become the predominate vaccine platform in the field (26, 27). In China, there are currently also two types of PRRS vaccines: MLV and KV vaccines. There are currently nine commercial PRRSV vaccines (CH-1a, CH-1R, VR2332/Ingelvac PRRS^®^ MLV, R98, JXA1-R, TJM-F92, HuN4-F112, GDr180, and PC). Of these, eight are MLV vaccines, VR2332 and R98 are of lineage 5, the others belong to lineage 8 (CH-1a, CH-1R, JXA1-R, TJM-F92, HuN4-F112, and GDr180) (20).

Since 2014 in China, an increasing number of studies have attempted to identify an available PRRS vaccine that can confer sterilizing or even effective heterologous-protection against the prevalent NADC30-like strain (26, 28–30). Unfortunately, none of the commercial vaccines on the market are ideal, which may be the reason that this strain escaped host immunity, spread quickly across the mainland, and rapidly became the dominant strain in China. However, most studies have found that MLV can provide partial protection in NADC30-like infected piglets, manifesting as a reduced titer and shorter duration of viremia, improved average daily weight gain (ADWG), and alleviation of the gross lesions in the lung (26, 28, 29). Such partial protection may be derived from the simultaneous triggering of cell-mediated-immunity (CMI) and humoral immunity.

To date, there have been few studies clarifying whether the KV vaccine can also deliver protection against NADC30-like strain. Previous studies indicate that KV cannot provide protection against PRRSV, particularly in PRRSV-naïve animals (31). In this study, we designed and evaluated a vaccination regimen in which piglets were primed with an MLV vaccine at 4 weeks of age and boosted with a KV vaccine at 7 weeks of age, respectively. In particular, we sought to determine whether a prime-boost regimen could induce robust antibody responses, increased ADWG, as well as decreased titer and duration of viremia, which would result in improved heterologous-protection against the NADC30-like strain. Furthermore, we aimed to determine whether the use of the KV vaccine as a booster could provide greater protection against the NADC30-strain over that of MLV vaccination alone.

## Materials and Methods

### Virus and MLV Vaccines

The NADC30-like PRRSV (v2016/ZJ/09-03) strain, isolated at the Asian Veterinary Research and Development Center [Boehringer Ingelheim (China) Investment Co., Ltd.] was used as the challenge virus (26, 28–30). Three PRRSV commercial vaccines were used in this study, including Ingelvac PRRS^®^ MLV (Boehringer Ingelheim), and another two domestic KV commercial vaccines.

### Animals and Experimental Design

The animal procedures used in this study were approved by the Ethics Committee for Animal Experimentation at Institute of Animal Health, Guangdong Academy of Agricultural Sciences. A total of 45 3-week-old, crossbred piglets were confirmed to be free of PRRSV antigens and antibodies using quantitative RT-PCR (qRT-PCR) (32) and a HerdChek PRRSX3 ELISA kit (IDEXX Inc.), respectively. Porcine circovirus type 2 (PCV2) and classical swine fever virus (CSFV) antigens were also tested negative using qRT-PCR (Beijing Anheal Laboratories Co., Ltd) for the presence of viral nucleic acids in the serum. All piglets were transported to animal facilities at the Guangdong Academy of Agricultural Sciences 1 week prior to integration and were subsequently randomly divided into five groups (Table 1) housed in five individual rooms. After cooling down for 1 week, piglets in the M1, MK1, and MK2 groups were intramuscularly immunized with a full dose of Ingelvac PRRS^®^ MLV at 4 weeks of age in accordance with the manufacturer's instructions. Piglets in the SC and PC groups were administered minimal essential medium (MEM) as a control. One piglet in the MK1 group was removed before the second vaccination due to severe bacterial infection.

**Table 1:** Experimental design.

**Group**	**1^**s*t***^ vaccination**	**2^**n*d***^ vaccination**	**NADC30-like strain challenge**	**Sacrifice**	**No. of piglets**
	**4 weeks of age**	**7 weeks of age**	**10 weeks of age**	**12 weeks of age**	
SC	MEM medium	MEM medium	MEM medium	Sacrificed	5
PC	MEM medium	MEM medium	10^4.5^ TCID_50_	Sacrificed	10
M1	Ingelvac PRRS^®^ MLV one dose	MEM medium	10^4.5^ TCID_50_	Sacrificed	10
MK1	Ingelvac PRRS^®^ MLV one dose	2 ml KV (A company)	10^4.5^ TCID_50_	Sacrificed	10-1^a^
MK2	Ingelvac PRRS^®^ MLV one dose	2 ml KV (B company)	10^4.5^ TCID_50_	Sacrificed	10

a *One piglet in group MK1 was culled before the 2nd vaccination because of severe bacterial infection*.

**Figure 1:**
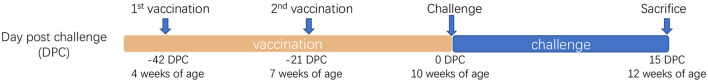
Time-line for each operation.

After 3 weeks, the piglets in the MK1 and MK2 groups received a second intramuscularly vaccination with 2 mL KV vaccines manufactured by domestic A and B companies, respectively. The piglets in the SC, PC and M1 groups were administered MEM in parallel. Three weeks following the second vaccination, piglets in SC group were given MEM medium, whereas the other groups were intramuscularly injected with 2 mL 4.5 Log^10^(TCID_50_) of v2016/ZJ/09-03 PRRSV. All pigs were euthanized and necropsied at 15 days post-challenge (DPC) (Figure 1).

### Sequence Similarity Analysis

The complete genomic sequences and open reading frames (ORF) 5 of commercial vaccine strains and v2016/ZJ/09-03 isolate used in this trial were aligned by using ClustalW in Lasergene software (DNASTAR Inc., Madison, USA). The representative PRRSV strains used for sequence similarity analysis were listed in (Table 2).

**Table 2:** Whole genome sequence (WGS) and ORF5 nucleotide identities between v2016/ZJ/09-03 with vaccine strains.

**Genome fragments**	**Isolates/** **brand**	**Ingelvac PRRS^®^ MLV (%)**	**Domestic company A (%)**	**Domestic company B (%)**
ORF5	v2016/ZJ/09-03	86.20	90.90	90.90
WGS	v2016/ZJ/09-03	87.10	88.50	88.50

### Serological Detection of Viremia

Two commercial ELISA kits were used for serological detection [IDEXX PRRS X3 Ab Test (IDEXX Laboratories, Inc., Westbrook ME, USA)], of antibodies specific for the PRRSV N protein. CIVTEST^®^ SUIS PRRS A/S PLUS (HIPRA, Girona, Spain) was used to detect antibodies for the PRRSV glycoprotein (GP) according to the manufacturer's instructions. PRRSV antibody results were reported as a sample value/positive value (S/P) ratio for the IDEXX kit and relative index × 100 (IRPC) for the HIPRA kit. Samples were considered positive if the S/P ratio was ≥0.4 and IRPC ≥ 20, respectively. Serological analysis was performed on a weekly basis from −42 DPC (4 weeks old, or the day of first vaccination) to 15 DPC (12 weeks old).

The level of viremia and viral shedding was tested using qRT-PCR as previously described (32). A PRRSV virus stock (v2016/ZJ/09-03) with a known titer of 6.2 Log^10^TCID_50_/mL was 10-fold serially diluted into virus-negative MEM medium, giving rise to theoretical infectious titers of 6.2 to −2.8 Log^10^TCID_50_/mL for the 10^0^-10^−10^ dilutions, respectively, with which a standard curve was generated [CT value = 3.2188 × Log^10^ (TCID_50_/reaction) + 29.65; *r*^2^ (correlation coefficient): 0.9977]. The cut-off value is 38 CT, and target gene is 3'UTR. The serum, nasal, and oral excretions were collected and tested at 0, 1, 6, and 15 DPC. Serum was collected to detect the level of viremia, and nasal and oral swabs were pooled together and used to indicate the magnitude of viral shedding.

### Virus Neutralization Test in Serum

Blood samples were collected on −42, −35, −28, −21, −14, −7, and 0 DPC. Virus neutralization test against challenge virus (v2016/ZJ/09-03) was performed in the serum as described previously (33). Briefly, serum samples were heat-inactivated at 56°C for 30 min, 100-μl serially diluted serum was mixed with an equal volume of v2016/ZJ/09-03 containing 200 TCID_50_. Each mixture was transferred to MARC-145 monolayers in 96-well plates after incubation 72 h at 37°C in an incubator containing 5% CO_2_. Cells were examined for cytopathic effects (CPE) with end-point titers as described previously (34, 35). Animals were considered to be protected when a titer of >8 (36).

### Clinical Assessment and Calculation of the Average Daily Weight Gain

Following challenge, the piglets were monitored daily by a clinical operator, and the clinical scores of each pig in all groups were recorded daily throughout the experiments. A detailed systemic scoring chart was employed with small modification as previously described (Supplementary Table 1) (37). The rectal temperature was tested and recorded daily following challenge. The number of average fever days in each group was counted and manifested on a group basis. Fever was defined if the animal's rectal temperature exceeded 40°C, and hyperpyrexia was defined as a rectal temperature above 41°C.

To avoid the subjective impact on the results, the animal husbandry staff were unaware of the vaccination status. In additional, average daily weight gain (ADWG) in each group were calculated based on the weight of each pig from 0 DPC to 15 DPC.

### Gross Pathological Examination of the Lung

At the end of the study period (15 DPC), all pigs were sacrificed and necropsied. The lungs were evaluated and scored based on the affected pulmonary lobe and the percentage of gross lung lesions using a standard scoring system for PRRS (38, 39).

### Examination of the Viral Load in the Lung, Hilar, and Inguinal Lymph Nodes

At 15 DPC, a piece of lung, hilar, and inguinal lymph nodes tissue randomly taken then marked from all sacrificed pigs. Tissues were modified to same weight for qRT-PCR analysis, as previously described (32).

### Statistical Analysis

Statistical analysis was carried out using GraphPad Prism 7.0 (GraphPad Software). The mean significance was determined for each comparison using a one-way analysis of variance with a Tukey's multiple comparison tests. A value *P* < 0.05 was considered statistically significant for differences between treatment groups.

## Results

### KV PRRS Vaccines Used as a Second Vaccination Could Not Further Stimulate Anti-N, or GP Protein Antibodies Generated in Response to a MLV Prime Vaccination Before Challenge

On day 1 of the study (-42 DPC), all piglets from each of the five groups were confirmed negative for PRRSV-specific antibodies and antigens. Prior to challenge (0 DPC), all piglets were clinically normal, demonstrating that the PRRS MLV vaccine has a high safety profile.

**Figure 2:**
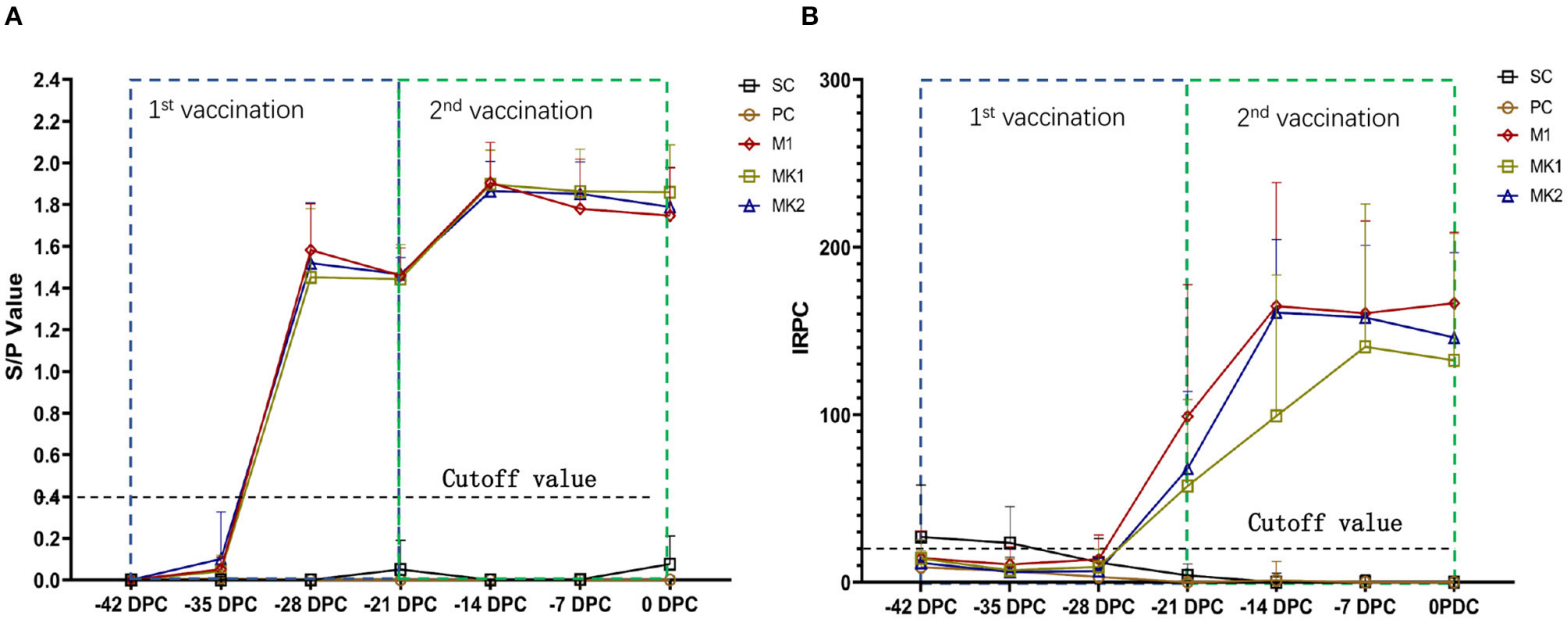
Serological reaction before challenge. **(A)** Anti-N protein antibody titer and data are expressed as mean SD (error bars); **(B)** Anti-GP proteins antibody titer and data are expressed as mean SD (error bars).

PRRSV anti-N protein antibodies were tested using an IDEXX ELISA kit following vaccination (Figure 2A). All piglets in the vaccinated groups displayed PRRSV seroconversion from 7 to 14 days and the antibody titer peaked at 28 days following the first vaccination. No significant differences were observed between M1 and MK groups regarding both the S/P value of each time point and changes in the antibody titers.

In addition, PRRSV anti-M and anti-GP protein antibodies were tested using an HIPRA ELISA kit following vaccination (Figure 2B). All piglets in the vaccinated groups displayed PRRS seroconversion from 14 to 21 days after the first vaccination. The anti-GP protein antibody titers in the M1 and MK2 groups peaked at 28 days after the first vaccination, 1 week earlier than that observed in the MK1 group. No significant differences in the antibody titer or kinetic trends were observed between the MK groups compared with singular MLV vaccination group. No anti-PRRSV-specific antibodies were detected in the piglets from the SC and PC groups prior to challenge. These results indicate that when used as a boost, immunization with KV PRRS vaccines could not further stimulate anti-N, nor GP protein antibodies, even after an MLV prime immunization.

Neutralizing antibodies (NAs) were not detected in any pigs in all groups before challenge (data not shown).

### MLV Vaccination Was Capable of Reducing Viremia and Viral Shedding Upon Challenge With the NADC30-Like Strain, but Could Not Be Further Enhanced by a Second KV Vaccination

**Figure 3:**
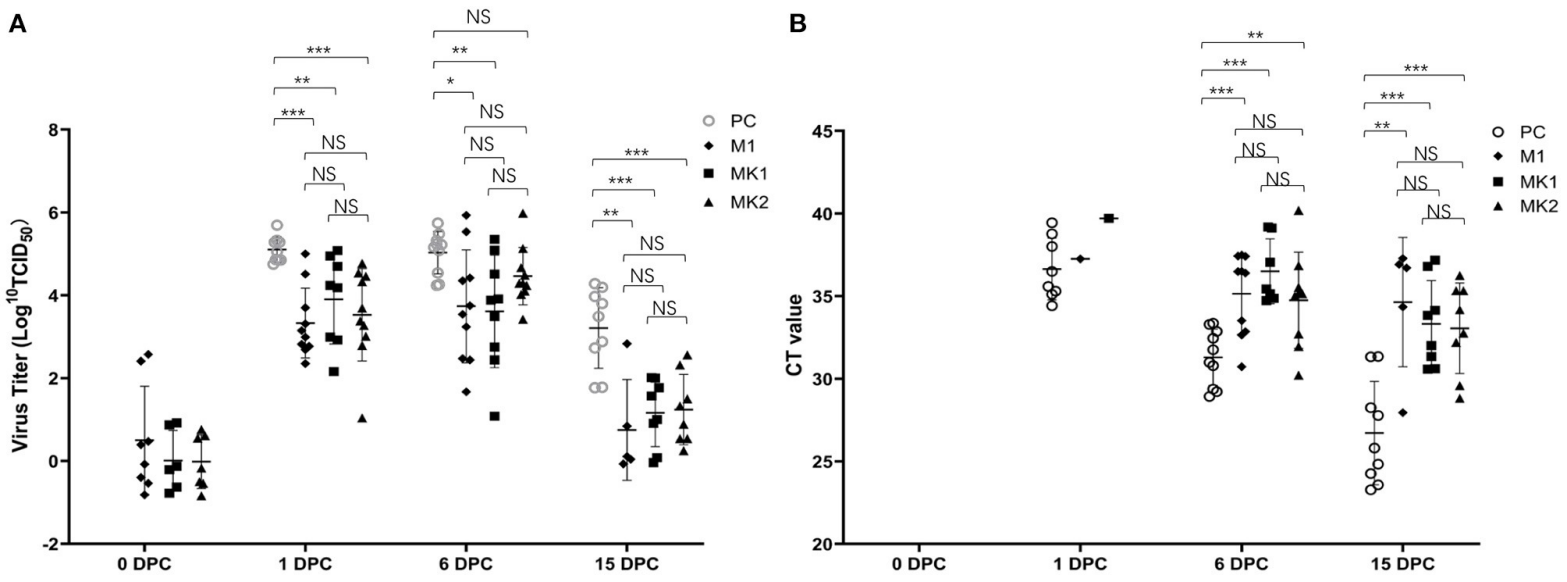
Viremia and virus shedding in the challenged pigs. **(A)** The Log^10^TCID_50_ and the number of viremia pigs were presented at 0, 1, 6, and 15 DPC. Data are shown as mean ± standard error (error bars). **(B)** The CT value and the number of virus shedding pigs were presented at 0, 1, 6, and 15 DPC. The sample was pooled by nasal and oral swab. Data are shown as mean ± standard error (error bars) (^*^p < 0.05; ^**^p < 0.01;^***^p < 0.001). NS, Not Statistically Significant.

Viremia was detected in the M1, MK1, and MK2 groups prior to challenge at 0 DPC. Following v2016/ZJ/09-03 PRRSV challenge, serum samples were collected from the piglets at 1 DPC, 6 DPC and 15 DPC to measure the level of viremia. As shown in Figure 3A, except the SC group, the level of v2016/ZJ/09-03 PRRSV viremia was detected in the PC, M1, MK1 and MK2 groups throughout the entire challenge period. However, not each pig after challenge was detected viremia positive at all timepoints especially at 15 DPC. The viremic strain was verified by ORF5 sequencing. At 1, 6 and 15 DPC, the viral loads in the piglets from the three vaccinated groups were lower than that of the PC group, except for the MK2 group at 6 DPC. These data indicate that the MLV vaccine reduced serum virus load in the challenged pigs. According to the timeline of viremia development, the viral titer increased slightly from 1 DPC, peaked at 6 DPC in the M1 and MK2 groups, and rapidly declined in all challenged groups. At 15 DPC, only 50% of the animals were detected to be positive for viremia in M1 group, compared to a 90% positive rate in the PC group. No viremia was detectable in the serum samples from the SC groups

Viral shedding was detected by a RT-PCR quantitative analysis in pooled oral and nasal swabs. None of the animals were RT-PCR positive prior to challenge at 0 DPC (Figure 3B). After the first day post-challenge (1 DPC), 50% of the animals in the PC group exhibited viral shedding, compared to 10% in M1 group, and 0% in the MK1, MK2 and SC groups. At 6 DPC, the positive rate increased to 100% in the PC and M1 groups, and 56 and 80% in the MK1 and MK2 groups, respectively. At the end of the study, the PC group slightly decreased to 90% positive rate, M1 group was decreased by 50% from 100%. And positive rate in MK1 increased to 89%. The positive rate in MK2 remains the same at both 6 and 15 DPC.

From the perspective of the viral shedding titer, the pigs in the PC groups shed a greater amount of virus compared to that of the MLV vaccination groups throughout the entire challenge period. The above data demonstrate that vaccination with MLV could decrease both the amount of virus spillover, as well as the number of shedding animals. Compared with the M1 group, there were no significant differences in the two MK groups regarding viral shedding, suggesting that KV vaccines are minimally effective against viral shedding.

### KV Vaccine Minimally Reduced the Duration of Fever and PRRS-Related Clinical Signs Compared With a Single MLV Vaccination

**Figure 4:**
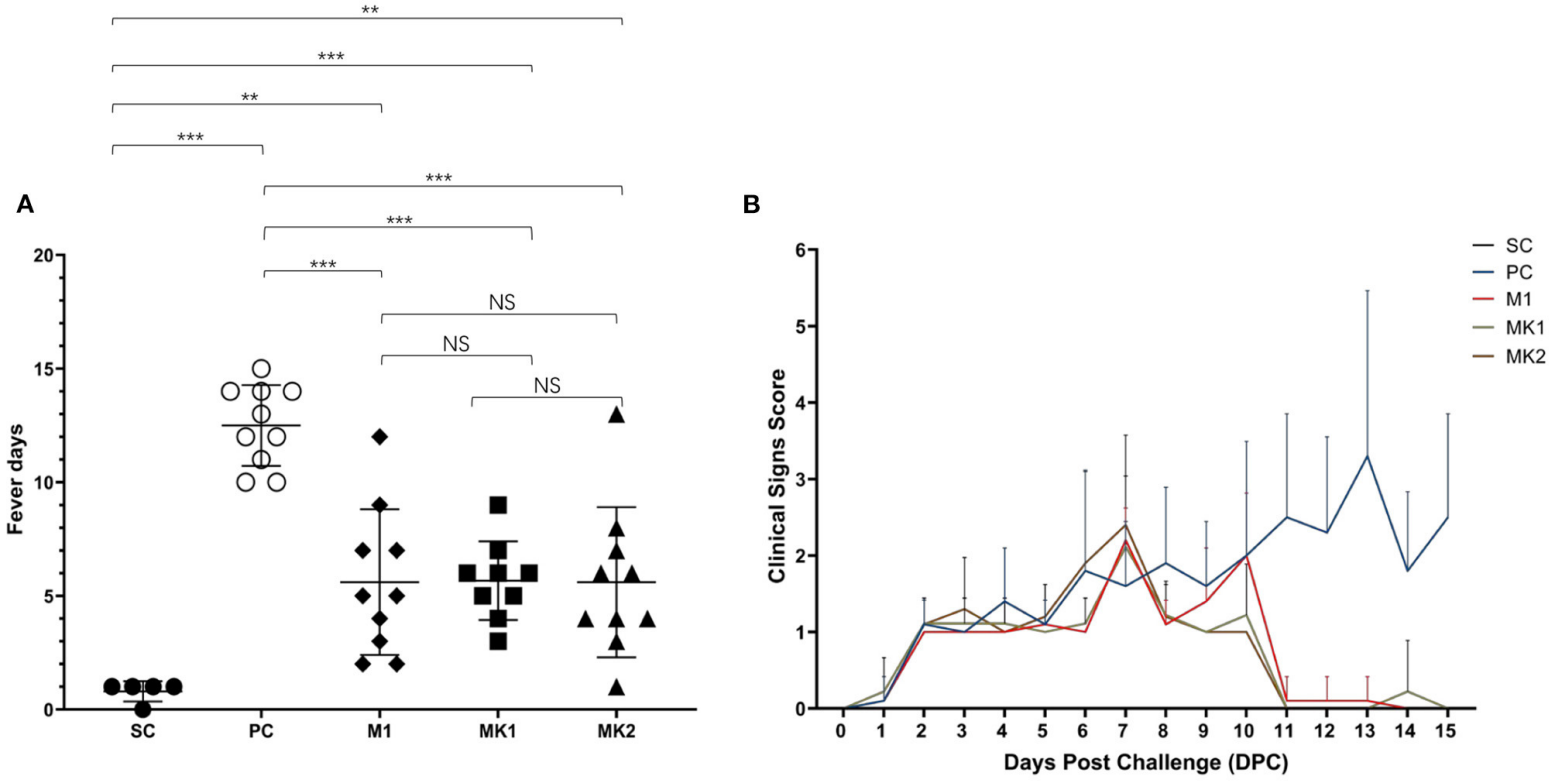
Average fever days and clinical sign scores after challenged. **(A)** Average fever days per group are shown as mean ± standard error (error bars). **(B)** The scores of clinical signs were added up included gross clinical score (GCS), respiratory clinical score (RCS) and nervous signs score (NSS), the data were shown as mean ± standard error (error bars) (^**^p < 0.01; ^***^p < 0.001). NS, Not Statistically Significant.

Throughout the entire challenge phase, none of the animals died due to PRRS infection. These studies indicate that v2016/ZJ/09-03 is not a highly virulent strain. In the SC group, 80% of the piglets had a one-day fever due to frequent operations, including body weight monitoring and sample collection at 0 DPC (Figure 4A). The average fever days was significantly lower in the piglets from the MLV vaccination groups compared to the pigs from the PC group (Figure 4A). Additionally, percentage of pigs experienced fever in each group at each time-point be analyzed and presented in (Table 3). These findings demonstrate that although MLV could not completely prevent the herd from fever, it could shorten the duration of pyrexia in the NADC30-like infected herd. Compared with the M1 group, there was no significant differences in the average fever days in the MK1 and MK2 groups, which indicated that KV vaccines exhibit a low efficacy toward alleviating fever duration.

**Table 3:** Percentage of feverous pigs in each group.

**Group**	**0 DPC (%)**	**1 DPC (%)**	**2 DPC (%)**	**3 DPC (%)**	**4 DPC (%)**	**5 DPC (%)**	**6 DPC (%)**	**7 DPC (%)**	**8 DPC (%)**	**9 DPC (%)**	**10 DPC (%)**	**11 DPC (%)**	**12 DPC (%)**	**13 DPC (%)**	**14 DPC (%)**	**15 DPC (%)**
SC	0	60	0	0	0	0	0	0	0	0	0	0	0	0	0	20
PC	0	100	30	50	70	100	100	90	90	100	100	90	90	70	80	50
M1	50	40	60	60	30	70	70	90	50	40	30	10	10	0	0	0
MK1	56	44	33	44	67	89	100	44	44	44	11	0	0	0	0	0
MK2	30	50	10	0	30	80	90	100	60	30	40	10	30	10	10	10

During the process from challenge to the end of the experiment, none of the piglets showed symptoms like nervous signs, lethargy, or cutaneous cyanosis. At 1 DPC, several pigs in all challenged groups began to exhibit PRRS-related clinical symptoms (e.g., inappetence). Beginning at 2 DPC, the number of pigs with inappetence gradually increased in the challenged groups, and some exacerbated from inappetence to a loss of appetite (Figure 4B). At 6 DPC, some pigs in the PC and MK2 groups began to display respiratory symptoms, such as a mild cough. Pigs in the M1 and MK1 groups showed symptoms (e.g., mild cough) at 7 DPC. The clinical symptoms of the pigs in the MLV immunization groups disappeared until 11 DPC. In contrast, the clinical symptom in PC group gradually became more severe (e.g., frequent cough and panting) (Figure 4B). Within the MLV vaccination groups, respiratory problems were observed in the MK2 group on 6 DPC, 1 day earlier than that of the M1 group. From 9 to 10 DPC, the overall clinical scores of the MK1 and MK2 groups were lower than that of the M1 group. Therefore, while MLV vaccination could improve PRRS-related clinical signs, there were no significant differences between the MK and M1 groups regarding the duration and severity of clinical PRRS disease.

### Pathological Lesions in the Lungs of Pigs Boosted With a KV Vaccine Are Not Further Reduced Upon v2016/ZJ/09-03 Challenge

**Figure 5:**
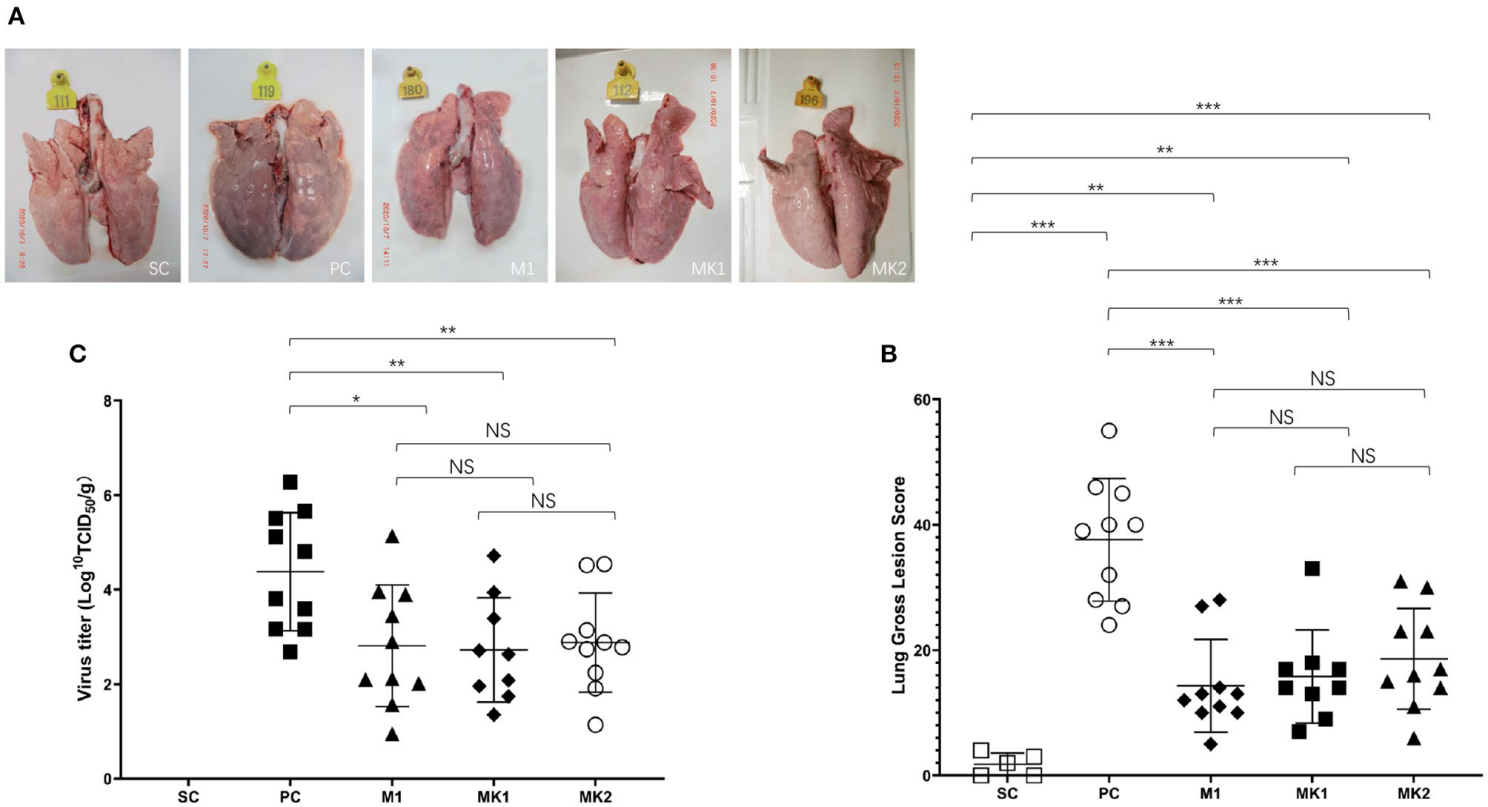
Gross lung lesion scores and virus load in lungs at 15 DPC. **(A)** Gross lung lesion examination. **(B)** Gross lung lesion scores were calculated based on the affected percentage of lung area, shown as mean ± standard error (error bars). **(C)** The virus load in lungs were measured at 15 DPC. Data were shown as mean ± standard error (error bars) (^*^p < 0.05; ^**^p < 0.01; ^***^p < 0.001). NS, Not Statistically Significant.

All of the challenged pigs survived until the end of the experiment before necropsy and quantification of gross lung lesions. Visible gross lung lesions were rarely observed in the pigs from the SC group (Figure 5A). Obvious lesions were observed in most of the pigs in the PC group, the affected lungs failed to collapse, and the parenchyma was firmer and heavier due to severe edema and congestion (Figure 5A). Mild to moderate interstitial pneumonia in the MLV vaccinated groups was primarily distributed in the cranial, middle, and ventromedial portion of the caudal lobes of the lungs (Figure 5A). Compared with the PC group, vaccinated groups had significantly lower gross lung lesion scores, whereas the difference was not significant between the MK and M1 groups according to the holistic gross lesion scoring system (Figure 5B).

The viral load degree in the lung seemed to have some correlation with the gross lesion severity present in the lung (Figure 5C). The viral load in the lungs of the pigs in the PC group was the highest, with an average of 4.4 Log_10_(TCID_50_)/g. The average viral load in the lungs of the MLV vaccination group was significantly lower than that in the PC group. No virus was detected in the SC group. These data show that the MLV vaccine could effectively reduce the viral load in v2016/ZJ/09-03-infected lungs by ~39.8 times. The viral load in the M1, MK1, and MK2 groups was 2.8, 2.7, and 2.9 Log^10^(TCID_50_)/g, respectively (Figure 5C), and there was no significant difference between the groups. These findings demonstrate that the KV vaccine could not eliminate virus from v2016/ZJ/09-03-infected lungs at 15 DPC.

Regarding to virus load in hilar and inguinal lymph nodes, there are no significant differences between PC and all vaccinated groups at 15 DPC (data not shown).

### ADWG Was Improved by ~400 g/Day in the MLV Vaccinated Pigs Compared With the PC Group From d0 to d15

**Figure 6:**
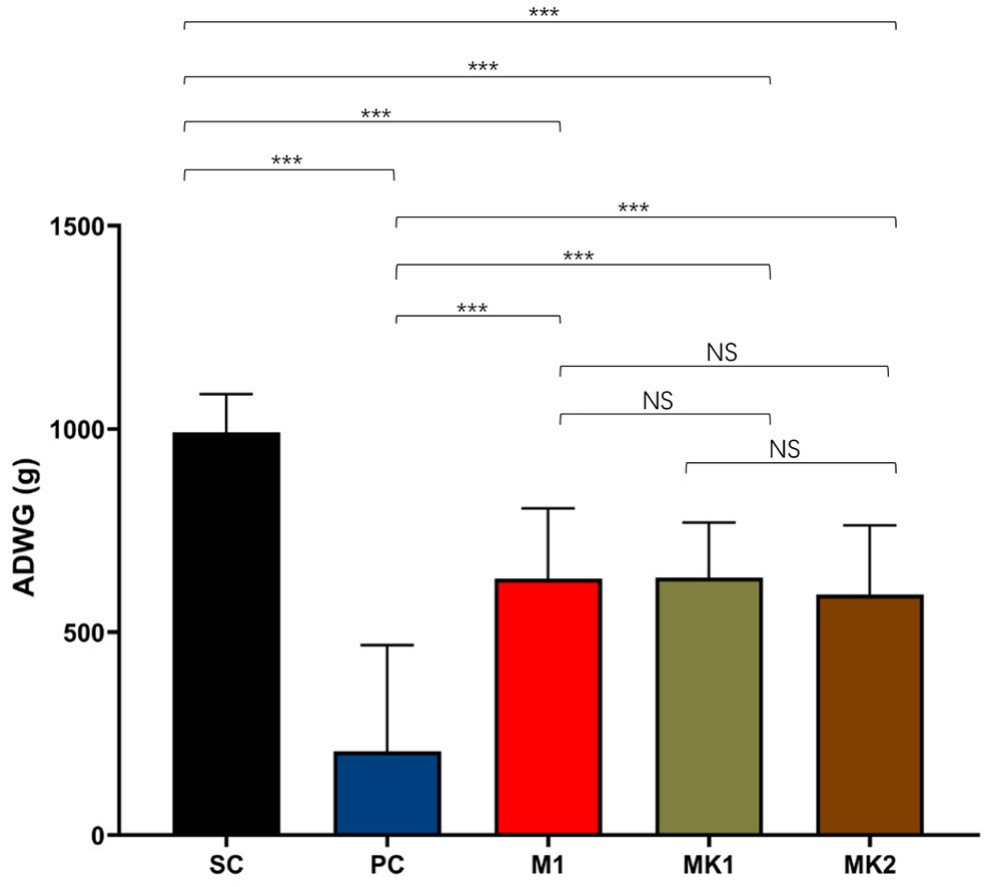
Average daily weight gain measurements. The average daily weight gain was calculated from 0 DPC to 15 DPC, shown as mean ± standard error (error bars) (^***^p < 0.001). NS, Not Statistically Significant.

The ADWG was calculated from the day of challenge (0 DPC) until the end of the experiment (15 DPC). Among all the groups, the ADWG of the SC group was the highest compared to that of the other groups. ADWG was the lowest in the PC group, followed by the MLV-vaccinated groups (Figure 6). These findings demonstrate that MLV vaccination is beneficial to the ADWG of challenged pigs. Consistent with the above data, there was no significant difference in ADWG between the MK and the M1 groups.

## Discussion

In 1995, PRRS was first identified and described in mainland China, and has subsequently caused substantial economic losses to the Chinese swine industry for the past 26 years (12). In 2014, NADC30-like strains began to dominate HP-PRRS and have since become the most prevalent strains in mainland China (13). According to one farm owner's description, the outbreak of PRRS due to NADC30-like infections in a farrow-finish farm with a 2,000-sow inventory resulted in over 2 million RMB losses in 2020. The Ingelvac PRRS^®^ MLV vaccine has been shown to provide cross-protection against over 10 genetically diverse PRRSV isolates, including several field-isolated NADC30-like strains currently circulating in China (26, 28–30, 40–42). However, compared with homologous protection, lower protection was observed in the heterologous challenge groups (19). KV vaccines represent an important tool for controlling viral infection, but the PRRS KV vaccine cannot stimulate a rapid humoral reaction, especially in naïve animals (43, 44). It was gradually applied as a booster shot in the field (45). To date, there have not been any available studies with respect to the protective efficacy of the PRRS KV vaccine against the NADC30-like strain. Therefore, in this study, a novel vaccination scheme was designed, in which naive piglets were first immunized with a PRRS MLV vaccine and boosted 3 weeks later with the PRRS KV vaccine. The aim of this study was to investigate the efficacy of a live-attenuated and inactivated PRRSV vaccine prime-boost regimen against a PRRSV NADC30-like strain in 4-week piglets.

An antibody ELISA represents one of the most stable and rapid methods used to monitor immune status in the field. In general, PRRS anti-N protein antibodies were seroconverted from day 7 to 14, and the anti-GP protein antibodies were initially detected from day 14 to 21 following live virus infection (46). Consistent with the results of previous studies, the same serological reactions were also reported in this study (47). No adverse reactions were observed in pigs throughout the entire MLV immunization process, indicating that the MLV vaccine has a good safety profile. However, mild to moderate side effects occurred less frequently following PRRS KV vaccination from domestic B Company, such as swelling and redness at the injection site.

In this study, compared with the M1 group, a booster with the KV vaccine 3 weeks after the first vaccine (MLV) could not stimulate higher levels of anti-N protein and anti-GP antibodies; the reasons for this finding can be as follows. First, for PRRSV, a classical anamnestic humoral response may not be the dominant response following a re-encounter with viral antigens (22, 48), or a 3-week interval may not be long enough for the MLV vaccine to stimulate B cells or differentiate memory B cell production. Secondly, the PRRSV N protein is a nucleocapsid protein that is covered and surrounded by the viral envelope (49). Moreover, the KV vaccine cannot replicate and express the N protein in host cells. Thus, KV vaccines manufactured by advanced purification technology may not produce the N protein in immunized animals. However, this cannot be used to explain why anti-GP protein antibodies could not be stimulated. Finally, the effective concentration of these two commercial KV vaccines were insufficient to activate the humoral immune response. From −21 to −14 DPC, anti-N protein antibodies increased simultaneously in all MLV-vaccinated groups, which may represent the normal serological reaction following MLV immunization (26, 28, 41) or may be caused by different batches of IDEXX ELISA kits that were used on −14 DPC and −21 DPC, respectively. From −42 to −35 DPC, the concentration of anti-GP5 protein antibodies in the SC group was greater than the cut-off value (20 IRPC) and became negative after −28 DPC, whereas the anti-N protein antibody was negative throughout the study. This may be caused by the presence of maternal antibodies in the piglets since anti-GP antibodies persist longer in the serum than anti-N protein antibodies (50).

It has been recognized that PRRSV viremia plays a key role in the development of respiratory diseases (42). An important parameter used to determine the efficacy of PRRSV vaccines is the reduction in PRRSV viral load in the blood (30, 51). Compared with the SC group, the MLV immunized groups could not completely prevent infection with the wild-type virus, as well as viral circulation in the blood. However, the viral load in the groups immunized with the MLV vaccine was reduced by 0.5 2.4 Log^10^ (TCID_50_) compared with the PC group. In addition, the proportion of positive pigs in the M1 group decreased to 50% at the end of the experiment. This finding may be due to the ability of MLV-immunized pigs to produce low neutralizing antibody titers, which is specific to wildtype virus strains as early as 5 days following wildtype virus infection (52). Thus, these antibodies may partially neutralize the virus present in the blood (52). Compared with the M1 group, the viral load in blood in the groups boosted with the KV vaccine did not further decrease during the entire challenge phrase. These findings were consistent with the results of the antibody tests. The inactivated vaccine could not stimulate the humoral immune response even after the first MLV vaccination, and consequently, could not reduce the viral load in the blood.

The susceptibility of pigs to PRRSV is related to the virulence of the strain, the magnitude of herd immunity, and amount of viral exposure in the environment (53). Therefore, it is critical for PRRS prevention and control to limit the degree of viral shedding in infected pigs (54). MLV-mediated reduction in viral shedding in the field was analyzed in this study as follows: (1) decrease in the amount of viral shedding by nearly 10 CT values at 15 DPC compared with the PC group; and (2) reduced shedding duration. Viral shedding was not detected in 50, 11, and 20% of pigs in the M1, MK1, and MK2 groups at 15 DPC, respectively. The virus positivity rate of the shedding animals was consistent with that exhibited by animals with viremia. This finding indicates that the observed viremia in this study was partially related to viral shedding within 15 days after infection. A higher viremia titer than viral shedding was observed on 1 DPC, which increased slightly until 6 DPC, then rapidly decreased. In contrast, the number of detected shedding animals was less than the number of viremia-positive animals on day 1 DPC, especially in the vaccinated groups. Moreover, the amount of viral shedding continued to increase throughout the entire challenge period. In summary, MLV could improve viral shedding in infected pigs, whereas KV could not.

A similar conclusion was achieved in a study showing that the severity of interstitial pneumonia was correlated with the viremia (55). In the PC group, gross lesions in the lung were the most severe and the viral load was the highest. Moreover, the affected lungs failed to collapse, and the parenchyma was firm and heavy due to severe edema and congestion. The severe respiratory failure symptoms observed in the PC group may be caused by the more severe gross lesion in the lungs. The viral load in the lung tissue of the MLV vaccination group was ~39.8 times lower than that of the PC group; however, the differences with or without intervention with the KV vaccine was minimal. This finding may be attributed to the fact that cell-mediated immunity is only minimally stimulated by the KV vaccine (56–58). Since MLV is a live attenuated virus, it can infect antigen-presenting cells, stimulate a cellular-mediated immune response, and initiate the apoptosis program of virally- infected cells (45). Virus present in apoptotic bodies can be quickly engulfed and degraded by professional or non-professional phagocytes that prevent virus spillover and re-infection of nearby cells. The KV vaccine is non-infectious, and prevention is derived from B cell recognition prior to activation of the humoral immune response; however, since it cannot infect antigen-presenting cells, it rarely stimulates the apoptosis reaction induced by cytotoxic T cells (56–58). Therefore, our findings show that while the MLV vaccine can effectively reduce the tissue viral load and alleviate gross lesions in the lung, the KV vaccine cannot.

Previous research has shown that the MLV vaccine can improve the ADWG of pigs after infection with heterologous PRRSV strains (26, 28–30, 40–42, 59). The ADWG in the PC group was only 206 g/d, which was 786 g/d lower than that in the SC group. However, compared with previous studies on HP-PRRS, the NADC30-like strain is less pathogenic and does not cause weight loss in unvaccinated pigs throughout the entire infection period (60). Compared with the SC group, MLV cannot completely protect pigs from the loss of ADWG caused by a PRRSV infection; however, compared with the PC group, the ADWG in MLV vaccinated pigs was ~600 g/d, which was the value of MLV for reducing the amount of economic loss in the field.

This study clarified vary vaccination schemes' heterologous-protection efficacy performance in PRRS naïve weaning piglets; however, in farrowing farms, sow herds have been historically more important for PRRS control and management (31). Therefore, different vaccination schemes and the associated protective efficacy should also be evaluated in sow herds by monitoring reproductive performance and serological reactions in the future.

## Conclusions

In conclusion, the data in this study demonstrate that although MLV cannot provide complete protection against an NADC30-like infection and prevent relevant clinical symptoms and gross lung lesions, it can reduce viral shedding and improve the ADWG following NADC30-like infection. It is important to quickly establish stability after infection with wild-type virus and reduce the economic losses caused by a PRRS outbreak.

In contrast, based on the above data, two commercial PRRS KV vaccines cannot provide protection against NADC30-like virus infection in naïve piglets within 15 days post-infection. There is no difference in the efficacy between a single injection with the MLV vaccine and MLV plus KV boost vaccination scheme. However, the booster injection with the KV vaccine directly increased the medication and labor cost, and indirectly increased the potential risk in biosecurity and animal welfare. Therefore, the data in this study show that the current domestic PRRS KV vaccines in China cannot provide adequate protected against the NADC30-like strain in infected piglets.

## Data Availability Statement

The original contributions presented in the study are included in the article/Supplementary Material, further inquiries can be directed to the corresponding authors.

## Ethics Statement

The animal study was reviewed and approved by the Ethics Committee for Animal Experimentation at Institute of Animal Health, Guangdong Academy of Agricultural Sciences, Permission Number: PT2020003.

## Author Contributions

KC, ZL, and CL designed the study. ZL, SF, YH, JQ, CL, and CZ conducted the experiments and managed all related data. ZS provide the v2016/ZJ/09-03 strain. CL analyzed the data and wrote the paper. JZ, NH, LH, and ZL checked and finalized the manuscript. All authors have read and approved the final manuscript.

## Funding

Boehringer-Ingelheim provided financial support for the conduct of the research. The purchase order is 4700148665.

## Conflict of Interest

CL, KC, JQ, YH, SF, LH, and NH were employed by Boehringer Ingelheim Animal Health (Shanghai) Co. Ltd. ZS was employed by Boehringer Ingelheim (China) Investment Co., Ltd. The remaining authors declare that the research was conducted in the absence of any commercial or financial relationships that could be construed as a potential conflict of interest.

## Publisher's Note

All claims expressed in this article are solely those of the authors and do not necessarily represent those of their affiliated organizations, or those of the publisher, the editors and the reviewers. Any product that may be evaluated in this article, or claim that may be made by its manufacturer, is not guaranteed or endorsed by the publisher.
