# Supplementary material for: Efficacy of the Synergy Between Live-Attenuated and Inactivated PRRSV Vaccines Against a NADC30-Like Strain of Porcine Reproductive and Respiratory Syndrome Virus in 4-Week Piglets
Source: Front Vet Sci. 2022 Feb 2;9:812040. doi: 10.3389/fvets.2022.812040 (PMC8847452; doi:10.3389/fvets.2022.812040)
Supplement: Supplementary file 1 [file Data_Sheet_1.PDF]

Supplementary Table 1. Clinical signs scoring system

	Clinical signs	Evaluation Criteria	Score
Gross clinical score (GCS)	1. Appetite	Normal	0
		Inappetence	1
		loss of appetite	2
	2. Awareness	Normal	0
		Lethargic	1
	3. Skin	Normal	0
		Rubefaction	1
Respiratory clinical score (RCS)	1. Respiratory condition	Normal	0
		Tachypnea (nervous)	1
		Tachypnea (calm)	2
		Tachypnea and dyspnea	3
		Tachypnea, dyspnea and Irregular respiration	4
	2. Cough	Normal	0
		Slight Cough (time/ half hour)	1
		Frequent cough (2-5 times/ half hour)	2
		Severe cough (More than 5 times/half hour)	3
Nervous signs score (NSS)	Nervous signs	Normal	0
		Shiver	1
		Ataxia	2
		Incoordination	3
		Paralysis	4

Usual condition: total score = GCS + RCS + NSS

If piglet died: total score = GCS + RCS + NSS + 5

$0 \leqslant$ total score $\leqslant 20$
